# Supplementary material for: mitoBKCa is functionally expressed in murine and human breast cancer cells and potentially contributes to metabolic reprogramming
Source: eLife. 2024 May 29;12:RP92511. doi: 10.7554/eLife.92511 (PMC11136494; doi:10.7554/eLife.92511)
Supplement: Figure 5—figure supplement 1—source data 1. [file elife-92511-fig5-figsupp1-data1.pdf]

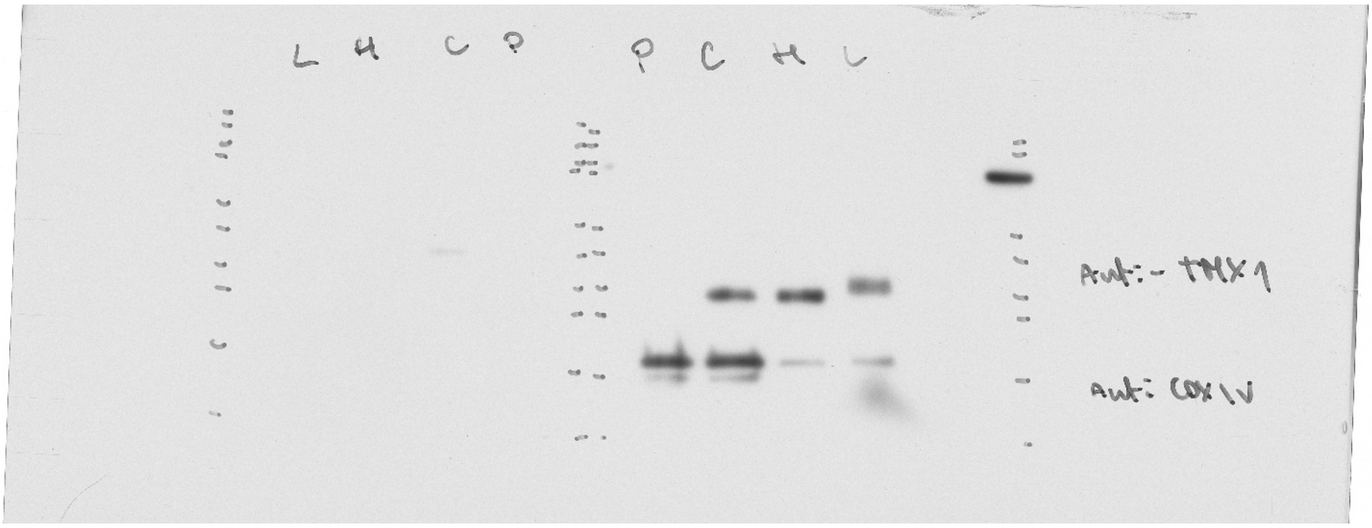
L H C P
P C H L
Ant:- TMX1
Ant: COX IV
