# Supplementary figures and images for: mitoBKCa is functionally expressed in murine and human breast cancer cells and potentially contributes to metabolic reprogramming
Source: eLife. 2024 May 29;12:RP92511. doi: 10.7554/eLife.92511 (PMC11136494; doi:10.7554/eLife.92511)

L H C P P C H L

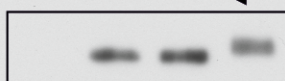

Anti: - TMX1

Anti: Cox IV

Supplement: Figure 5—figure supplement 1—source data 2. [file elife-92511-fig5-figsupp1-data2.pdf]

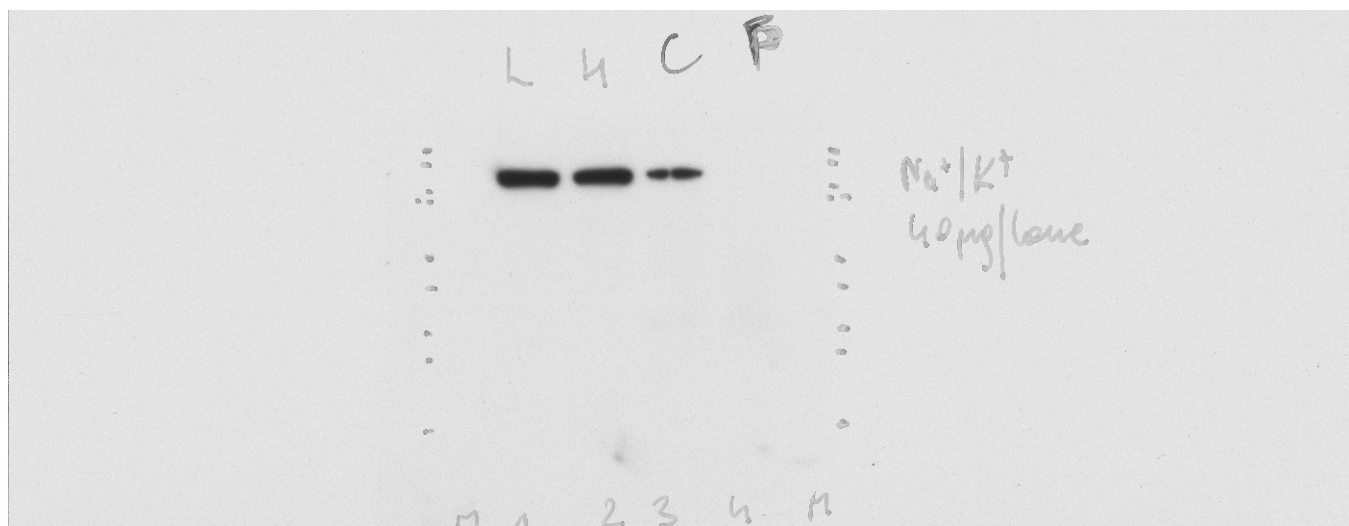

Supplement: Figure 5—figure supplement 1—source data 3. [file elife-92511-fig5-figsupp1-data3.pdf]

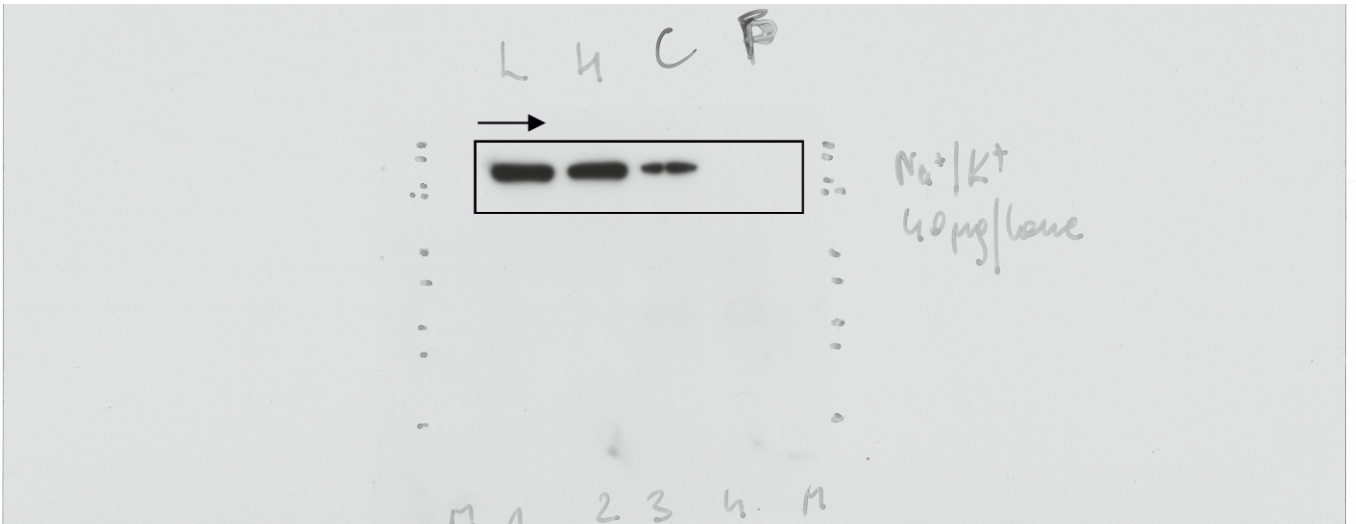

Supplement: Figure 5—figure supplement 1—source data 4. [file elife-92511-fig5-figsupp1-data4.pdf]

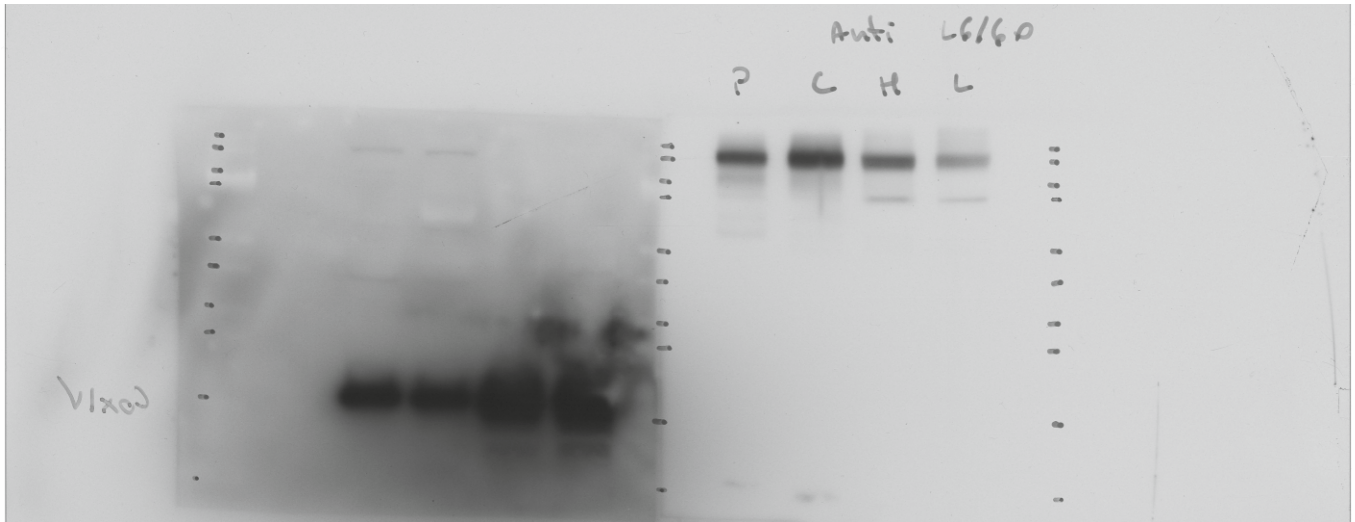

Supplement: Figure 5—figure supplement 1—source data 5. [file elife-92511-fig5-figsupp1-data5.pdf]

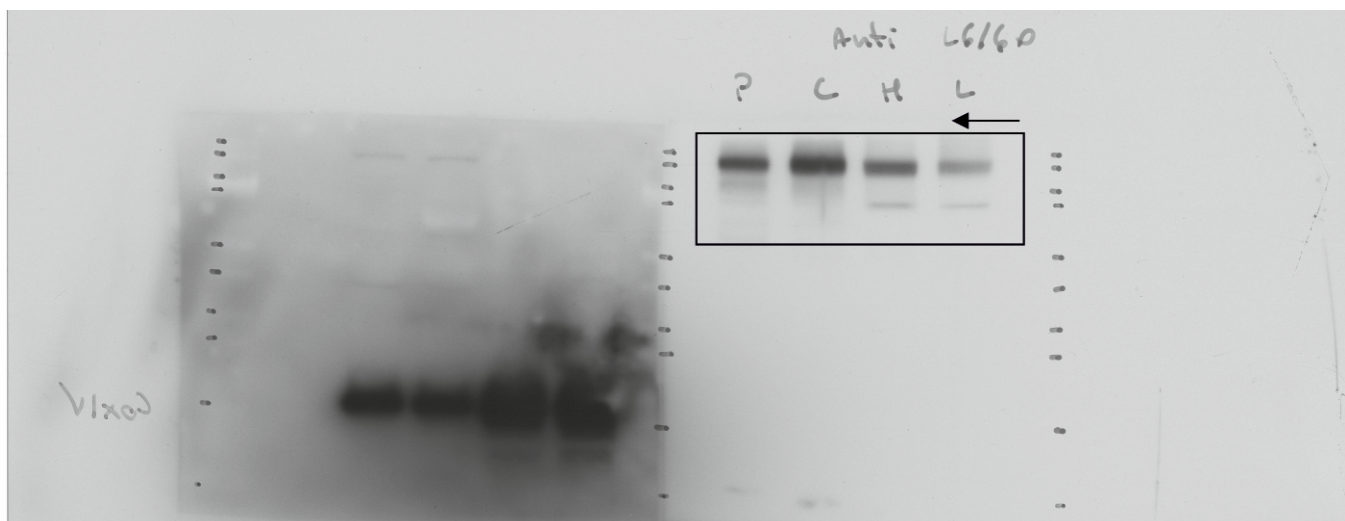

Supplement: Figure 5—figure supplement 1—source data 6. [file elife-92511-fig5-figsupp1-data6.pdf]

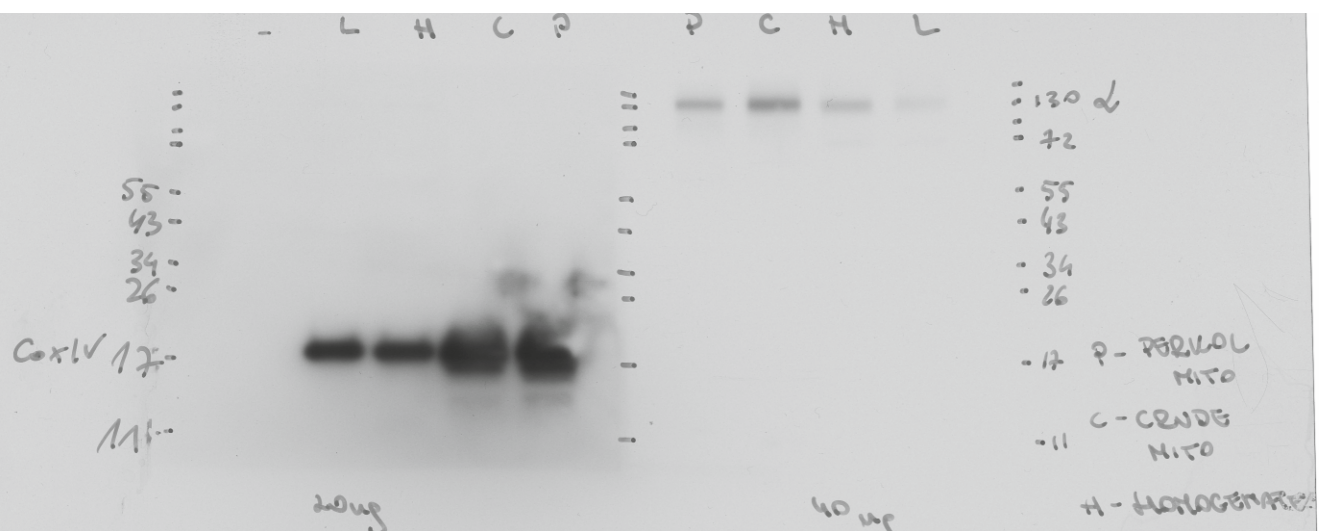

Supplement: Figure 5—figure supplement 1—source data 7. [file elife-92511-fig5-figsupp1-data7.pdf]

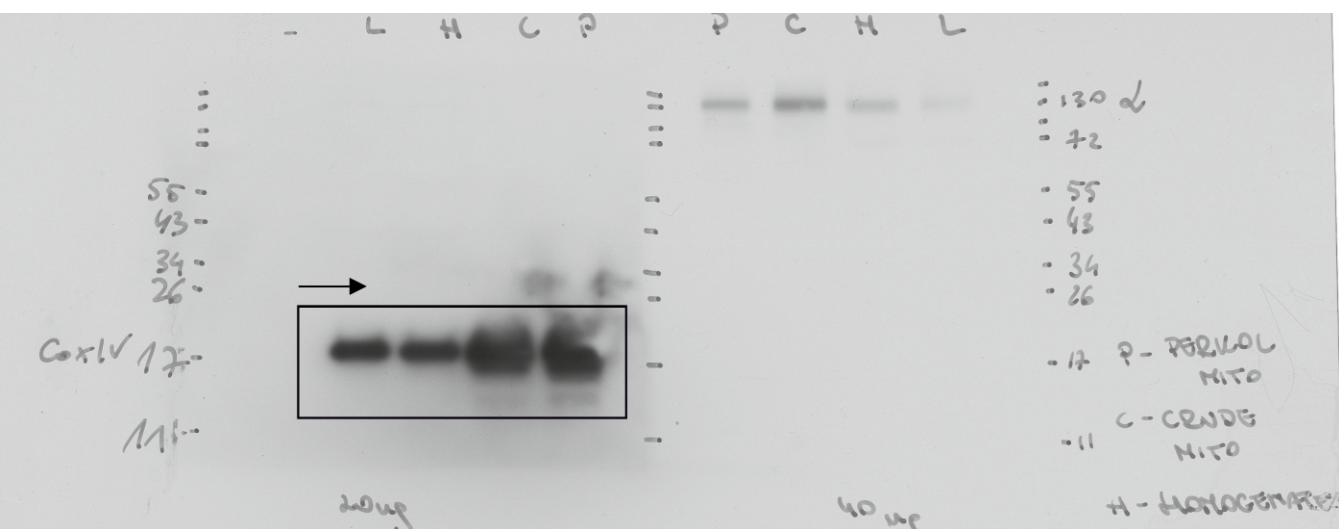

Supplement: Figure 5—figure supplement 1—source data 8. [file elife-92511-fig5-figsupp1-data8.pdf]
