# Supplementary material for: mitoBKCa is functionally expressed in murine and human breast cancer cells and potentially contributes to metabolic reprogramming
Source: eLife. 2024 May 29;12:RP92511. doi: 10.7554/eLife.92511 (PMC11136494; doi:10.7554/eLife.92511)
Supplement: Supplementary file 1. — (a) Probes used for Nanostring nCounter gene expression analysis. (b) Primers used for qPCR analysis. (c) siRNAs used for silencing based experiments. [file elife-92511-supp1.docx]

**File 1a**

| **Gene** | **Accession number** | **Target sequence (5’ – 3’)** |
| --- | --- | --- |
| ABCF1 | NM_001090.2 | TCCCGCCAAGCCATGTTAGAAAATGCATCTGACATCAAGCTGGAGAAGTTCAGCATCTCCGCTCATGGCAAGGAGCTGTTCGTCAATGCAGACCTGTACA |
| NRDE2 | NM_017970.3 | TGGAGGTCCTATGTACAGATTCAGAATAAGTCCCACAGTGCCAGCAAAACCAGGAGATTTTTTGACACAATCACCAGGTCTGCCAAACCCTTGGAGCCTT |
| POLR2A | NM_000937.2 | TTCCAAGAAGCCAAAGACTCCTTCGCTTACTGTCTTCCTGTTGGGCCAGTCCGCTCGAGATGCTGAGAGAGCCAAGGATATTCTGTGCCGTCTGGAGCAT |
| PUM1 | NM_001020658.1 | CTGGGGAACATCAGATCATTCAGTTTCCCAGCCAATCATGGTGCAGAGAAGACCTGGTCAGAGTTTCCATGTGAACAGTGAGGTCAATTCTGTACTGTCC |
| SF3A1 | NM_005877.4 | GATGATGAGGTGTACGCACCAGGTCTGGATATTGAGAGCAGCTTGAAGCAGTTGGCTGAGCGGCGTACTGACATCTTCGGTGTAGAGGAAACAGCCATTG |
| KCNMA1 | NM_001014797.2 | CCGTGCGACAGCCGGGGCCAACGCATGTGGTGGGCTTTCCTGGCCTCCTCCATGGTGACTTTCTTCGGGGGCCTCTTCATCATCTTGCTCTGGCGGACGC |
| KCNMA1-DEC | XM_024447988.2 | AAACAGAATGCAACAAGGATGAATAGAATGGGCCAAGAAAAGAAATGGTTTACAGATGAACCGGATAATGCCTATCCCAGAAACATTCAAATCAAGCCCA |

**File 1b**

| **Primer name** | **Sequence (5’ – 3’)** | **Species** | **Amplicon size** |
| --- | --- | --- | --- |
| BK_Ca_-DEC for | CAAACAGAATGCAACAAGGATG | Human / mouse | 124 bp |
| BK_Ca_-DEC rev | GTTAGCCATGTGGGTACTC | Human / mouse |  |
| BK_Ca_ for | CGCCTCTTCATGGTCTTC | Human / mouse | 134 bp |
| BK_Ca_ rev | ATGTGCTTTCTTCCACTAAC | Human / mouse |  |
| h β-tubulin for | GGCCAGATCTTTAGACCAGAC | Human | 120 bp |
| h β-tubulin rev | CACATCCAGGACAGAATCAAC | Human |  |
| m β-tubulin for | AGTGTGGCAACCAGATC | Mouse | 114 bp |
| m β-tubulin rev | AGTAAACGCTGATCCTCTC | Mouse |  |

**File 1c**

| **siRNA name** | **Sequence (5’ – 3’)** | **Targeted Species** |
| --- | --- | --- |
| siScrbl | UUCUCCGAACGUGUCACGU-dTdT | Human / mouse |
| siBK | UAGGAAACCGCAAGAAAUA-dTdT | Human / mouse |
| siBK-DEC | CCAGAUCAACCAAUAUAAA-dTdT | Human / mouse |
